# Effect of handgrip on coronary artery disease and myocardial infarction: a Mendelian randomization study

**DOI:** 10.1038/s41598-017-01073-z

**Published:** 2017-04-19

**Authors:** Lin Xu, Yuan Tao Hao

**Affiliations:** 1 School of Public Health, Sun Yat-sen University, Guangzhou, Guangdong Province China; 2 School of Public Health, Li Ka Shing Faculty of Medicine, The University of Hong Kong, Hong Kong, SAR China

## Abstract

Observational studies have reported an association of handgrip strength with risk of cardiovascular disease. However, residual confounding and reverse causation may have influenced these findings. A Mendelian randomization (MR) study was conducted to examine whether handgrip is causally associated with cardiovascular disease. Two single nucleotide polymorphisms (SNPs), *rs3121278* and *rs752045*, were used as the genetic instruments for handgrip. The effect of each SNP on coronary artery disease/myocardial infarction (CAD/MI) was weighted by its effect on handgrip strength, and estimates were pooled to provide a summary measure for the effect of increased handgrip on risk of CAD/MI. MR analysis showed that higher grip strength reduces risk for CAD/MI, with 1-kilogram increase in genetically determined handgrip reduced odds of CAD by 6% (odds ratio (OR) = 0.94, 95% confidence interval (CI) 0.91–0.99, P = 0.01), and reduced odds of MI by 7% (OR = 0.93, 95% CI 0.89–0.98, P = 0.003). No association of grip strength with type 2 diabetes, body mass index, LDL- and HDL-cholesterol, triglycerides and fasting glucose was found. The inverse causal relationship between handgrip and the risk of CAD or MI suggests that promoting physical activity and resistance training to improve muscle strength may be important for cardiovascular health.

## Introduction

Handgrip strength, a prognostic marker for healthy aging, has been associated with a number of chronic disease outcomes in observational studies. Specifically, greater grip strength was associated with lower risks of diabetes^1^, metabolic syndrome^2^, cardiovascular disease and mortality^3^. However, observational studies on grip strength may be subject to residual confounding such as body size and underlying illnesses, and reverse causality. Grip strength is well correlated with measures of body size especially body mass index, and also reflects functional capacity and frailty^4^, which could be affected by chronic disease, malnutrition, falls and hospitalization in older people. Traditional observational studies cannot account for all possible confounders. Thus, it is unclear whether grip strength, as a marker of muscle strength, causes the metabolic abnormalities or cardiovascular disease per se, or is only a predictor of underlying health conditions.

A large randomized controlled trial (RCT) on resistance training to improve grip strength with cardiovascular events as the primary endpoint would be definitive, but will take several years and might be difficult to conduct because of poor compliance. Moreover, whether the effects, if any, are due to the improvement in grip strength or other intervention efforts (i.e. changes in diet) is unclear. Mendelian randomization (MR) studies make use of genetic variants as instrumental variables to investigate the effect of environmental exposures on health outcomes. Since alleles are randomly allocated after conception and do not change during lifetime, MR studies are less vulnerable to confounding from non-genetic factors and to reverse causality. Thus it can be used to infer causality as further extensions to observational studies^5^. Many MR studies have been successfully conducted in cardiovascular research to investigate potential etiological mechanisms, prioritize drug targets and increase understanding of current therapies^6^.

Here, single nucleotide polymorphisms (SNPs) identified in a recent genome wide association study (GWAS)^7^ were used as genetic instrumental variables, to examine the causal effect of handgrip on coronary artery disease (CAD) and cardiovascular risk factors.

## Methods

### Data sources

#### Genetic instrumental variable for handgrip

From the most updated GWAS from the CHARGE (Cohorts for Heart and Aging Research in Genomic Epidemiology Consortium) consortium on handgrip, 2 SNPs [i.e. rs3121278 in *BMS1L* (BMS1-like ribosome biogenesis protein) and rs752045 in *CSMD1* (CUB and Sushi multiple domains 1)] independently contributing to grip strength (kg) at genome wide significance level (p < 5 * 10^−8^) in the discovery stage were used as genetic instrumental variables in the Mendelian randomization analysis (Table 1)^7^. To fully take advantage of all data available in the CHARGE, statistics of these two SNPs were obtained from the combined discovery and replication set. The pleiotropic effects of these 2 SNPs were identified from Ensembl (Homo sapiens – phenotype) (http://grch37.ensembl.org/Homo_sapiens/Info/Index), a comprehensive genotype to phenotype cross-reference. As no other phenotypes were reported for these 2 SNPs except for handgrip, indicating pleiotropy was unlikely, both of them were included in the current analysis.

**Table 1:** Characteristics of the SNPs^†^ used as genetic instrumental variables of handgrip strength (kg).

Nearest gene	SNP	Effect (beta)^‡^	SE	Effect allele	Other allele	P-value	EAF	Sample size
BMS1L	rs3121278	−0.26	0.06	T	G	6.18e-5	0.18	34,910
CSMD1	rs752045	0.47	0.08	G	A	5.20e-10	0.18	34,910

BMS1L: BMS1-like ribosome biogenesis protein; CSMD1: CUB and Sushi multiple domains 1; SNP: single-nucleotide polymorphisms; SE; standard error; EAF: effect allele frequency.

^‡^Effect on handgrip per kilogram per copy of the effect allele.

^†^All information was obtained from “Matteini, A. M. *et al*. GWAS analysis of handgrip and lower body strength in older adults in the CHARGE consortium. Aging Cell, doi:10.1111/acel.12468 (2016)”.

### Coronary artery disease and its risk factors

Association of SNPs with the phenotypes were extracted from publicly available consortia. Data on coronary artery disease/myocardial infarction have been contributed by Coronary ARtery DIsease Genome wide Replication and Meta-analysis (CARDIoGRAM) plusC4D investigators and have been downloaded from www.CARDIOGRAMPLUSC4D.ORG^8^. The summary data on the gene-CAD association were obtained from the CARDIoGRAMplusC4D 1000 Genomes-based GWAS, a meta-analysis of GWAS studies of mainly European, South Asian, and East Asian, descent imputed using the 1000 Genomes phase 1 v3 training set with 38 million variants^9^. The study interrogated 9.4 million variants and involved 60,801 coronary artery disease (CAD) cases and 123,504 controls, and 43,676 myocardial infarction (MI) cases and 128,199 controls^9^. Data on T2DM was contributed by the DIAbetes Genetics Replication And Meta-analysis (DIAGRAM, http://diagram-consortium.org/downloads.html), which includes 12,171 cases and 56,862 controls in Stage 1 GWAS^10^ and 26,488 cases and 83,964controls in the Trans-ethnic GWAS meta-analysis^11^. Genetic associations with BMI (kg/m^2^) have been contributed by The Genetic Investigation of ANthropometric Traits (GIANT) investigators and have been downloaded from https://www.broadinstitute.org/collaboration/giant/index.php/GIANT_consortium_data_files which has BMI for 152,893 men and 171,977 women of European ancestry^12^.

Genetic associations with high density lipoprotein (HDL) cholesterol, low density lipoprotein (LDL) cholesterol, triglycerides, and total cholesterol in 188,577 people have been contributed by Global Lipids Genetics Consortium (GLGC) investigators and have been downloaded from http://csg.sph.umich.edu/abecasis/public/lipids2013/ ^13^. Genetic associations with fasting insulin (n = 38,238) and fasting glucose (n = 46,186) have been contributed by Meta-Analyses of Glucose and Insulin-related traits Consortium (MAGIC) investigators and have been downloaded from http://www.magicinvestigators.org/, which relates to people of European ancestry without diabetes^14^.

### Statistical analysis

SNP-specific Wald estimates (ratio of SNP on outcome to SNP on handgrip) of the effect of handgrip on each outcome were combined using inverse-variance weighted (IVW) method giving an odds ratio (OR) for CAD and MI, and beta coefficients (log odds ratio of CAD/MI per 1 kg greater handgrip) for the other outcomes with 95% confidence interval (CI), based on the following formulas^15^:

1 $${\hat{\beta }}_{IVW}=\,\frac{{\sum }_{K=1}^{K}{E}_{k}{D}_{k}{\sigma }_{Dk}^{-2}}{{\sum }_{K=1}^{K}{E}_{k}^{2}{\sigma }_{Dk}^{-2}}$$

2 $$S{E}_{{\hat{\beta }}_{IVW}}=\sqrt{\frac{1}{{\sum }_{k=1}^{k}{E}_{K}^{2}{\sigma }_{Dk}^{-2}}}$$

where E_K_ is the mean change in exposure level (grip strength) per additional effect allele of SNP k and D_k_ is the mean change in disease outcomes (e.g. log odds of CAD or levels of other CVD risk factors) per additional effect allele of SNP k with standard error σ_Dk_. The weakness of the instruments was evaluated using the first-stage F-statistics calculated by

$$F=\frac{{R}^{2}/K}{(1-{R}^{2})/(n-K-1)},$$

where R^2^ indicates the variance explained by each genetic instrument, K indicates the number of instrument, and n indicates the sample size of the first stage^16^. The R^2^ of each SNP was calculated using the effect allele frequency (f) and beta (β) from the results of the CHARGE consortium using the following formula^12^: R^2^ = β^2^ * (1 − f) * 2f. Statistical analysis was performed using STATA 14.0.

## Results

The first-stage F-statistics for the IV including these 2 SNPs was 128. Tables 1 and 2 show the associations of 2 handgrip-associated SNPs, used as genetic instrumental variables in the Mendelian randomization analysis, with grip strength levels and CAD risk. Each handgrip increasing allele was associated with 5–7% reduction in CAD risk (OR 0.93, 95% CI 0.85–1.02 for rs3121278, and OR 0.95, 95% CI 0.9, 0.9995 for rs752045) and 6–10% reduction in MI risk (OR 0.90, 95% CI 0.81–0.99 for rs3121278 and OR 0.94, 95% CI 0.89–0.99 for rs752045). No association was found for type 2 diabetes, body mass index, HDL-cholesterol, LDL-cholesterol, triglycerides and fasting glucose. Table 3 shows that each kilogram increase in handgrip strength decreased CAD risk by 6% (odds ratio (OR) 0.94, 95% CI 0.91 to 0.99) and MI risk by 5% (OR 0.93, 95% CI 0.89 to 0.98).

**Table 2:** Odds ratio (95% confidence interval) for coronary artery disease, myocardial infarction and type 2 diabetes, and mean difference (standard error) of cardiovascular risk factors per allele of SNPs used in Mendelian randomization analyses.

SNP	rs3121278		rs752045	
	**OR (95% CI)**	**P-value**	**OR (95% CI)**	**P-value**
Coronary artery disease	0.93 (0.85, 1.02)	0.12	0.95 (0.9, 0.9995)	0.047
Myocardial infarction	0.90 (0.81, 0.99)	0.03	0.94 (0.89, 0.99)	0.03
Type 2 diabetes	0.97 (0.86, 1.08)	0.57	1.00 (0.93, 1.08)	1.00
	**Mean difference (SD)**	**P-value**	**Mean difference (SD)**	**P-value**
Body mass index, SD^†^	0.99 (0.96, 1.02)	0.50	1.01 (0.98, 1.03)	0.62
LDL-cholesterol, SD^†^	0.99 (0.96, 1.03)	0.63	1.00 (0.97, 1.03)	0.88
HDL-cholesterol, SD^†^	1.02 (0.98, 1.05)	0.39	0.98 (0.96, 1.01)	0.19
Triglycerides, SD^†^	0.99 (0.96, 1.02)	0.52	1.00 (0.97, 1.02)	0.74
Fasting glucose, mmol/l	0.99 (0.97, 1.02)	0.62	1.00 (0.98, 1.02)	0.92

SNP: single-nucleotide polymorphisms; OR: odds ratio; SD: standard deviation; LDL: low density lipoprotein; HDL: high density lipoprotein;

^†^1-SD equals to 4.5 kg/m^2^ for BMI, 38.7 mg/dL for LDL-cholesterol, 15.5 mg/dL for HDL-cholesterol, and 90.7 mg/dL for triglycerides.

**Table 3:** Causal effect of handgrip strength (kg) on cardiovascular risk factors, diabetes and coronary artery disease.

	Odds ratio	95% confidence interval	p-value
Coronary artery disease	0.94	0.91 to 0.99	0.01
Myocardial infarction	0.93	0.89 to 0.98	0.003
Type 2 diabetes	0.99	0.96 to 1.02	0.52
	**Beta**	**95% confidence interval**	**p-value**
Body mass index, SD^†^	0.0003	−0.01 to 0.02	0.97
LDL-cholesterol, SD^†^	−0.005	−0.01 to 0.001	0.11
HDL-cholesterol, SD^†^	−0.002	−0.03 to 0.02	0.90
Triglycerides, SD^†^	−0.007	−0.03 to 0.01	0.49
Fasting glucose, mmol/l	−0.003	−0.01 to 0.0006	0.09

^†^1-SD equals to 4.77 kg/m2 for BMI, 38.7 mg/dL for LDL-cholesterol, 15.5 mg/dL for HDL-cholesterol, and 90.7 mg/dL for triglycerides.

## Discussion

The current Mendelian randomization analysis using the most updated GWAS results on handgrip found that greater grip strength was significantly associated with lower risks of coronary artery disease and myocardial infarction. Moreover, greater handgrip tends to be associated with more-favorable cardiovascular disease biomarkers, including LDL-cholesterol and triglycerides, although the result was not statistically significant in the MR analysis. The MR analysis did not support a causal effect of handgrip on body mass index or HDL-cholesterol.

No randomized controlled trial (RCT) specifically on handgrip was found. One recent RCT on resistance training showed that higher-volume resistance training improved muscle strengths and also reduced LDL-cholesterol^17^. However, as levels of some inflammation markers such as interleukin-1 and interleukin-6 were also reduced during the resistance training, whether the beneficial effect on cardiometabolic health was due to the improvement in muscular strength or the reduction in inflammation was unclear^17^. A previous review of physiologic research also suggested functional and metabolic benefits of muscle strength, including potential causal pathway^18^. Moreover, results of the current MR analysis are in line with earlier observational studies showing that greater grip strength in adulthood was associated with lower risks of cardiovascular mortality, irrespective of sex and age groups^19, 20^. However, in traditional observational studies, the beneficial effects could be confounded by general physical fitness, which was associated closely with both muscle strength and the risk of cardiovascular disease^21^. Individuals with lower muscular strength may also be less healthy overall than those with higher grip strength, while those with higher grip strength may also have more leisure time physical activity and tend to participate resistance training^22^. Thus, as a method that complementary to and analogous with RCTs, Mendelian randomization may be an appropriate study design to assess whether muscle strength can directly affect risk of cardiovascular diseases. Already, regarding the long-held candidates of CAD biomarkers, such as LDL-cholesterol^23^, HDL-cholesterol^24^, blood pressure^25^ and glycosylated hemoglobin A1c^26^, MR has been suggested to be an useful approach to infer causality^27^.

To date, no Mendelian randomization on handgrip was found. The current study is the first Mendelian randomization study providing causal evidence in terms of a protective effect of handgrip strength on the CAD/MI risk. The strengths of this study include the very large sample size and the use of genetic variants to avoid some of the key limitations of traditional multivariable regression approaches. Mendelian randomization study using a small number of genetic variants in specific gene regions as instrumental variable will provide close parallels to a RCT^28^. As in two-sample MR, data on phenotype and outcomes can be obtained from different individuals, genetic associations with the phenotype and outcomes can be estimated on large consortia, thus it greatly increases power compared with Mendelian randomization analysis in one sample^15^. Moreover, compared with MR within one sample which is more likely to subject to weak instrumental bias due to potential correlation between genetic variants and confounders, two-sample MR may avoid statistical overfitting but tends to provide conservative estimation^29^.

Several assumptions or methodologic considerations bear discussion. First, both genetic variants used for genetically determined handgrip were strongly related to handgrip. No obvious reason exists for the existence of confounders of the association between the genetic variants and the outcomes considered here, for example by population stratification, because the underlying studies relate to relatively ethnically homogeneous populations of mainly European ancestry. Second, the genetic variants used are not known to be associated with other phenotypes that might influence coronary artery disease and or risk factors, thus making biases from direct associations of SNPs with the outcomes, i.e., “pleiotropy” or violation of the “exclusion-restriction” assumption, unlikely. Moreover, we found no evidence of horizontal pleiotropy, i.e. that the genetic variants used to predict handgrip had effects on coronary artery disease or its risk factors independent of effects via handgrip. Third, given the use of summarized data in two samples, handgrip was not measured in the sample with the outcome. However, two-sample instrumental variable analysis is less vulnerable to more robust to chance associations than analysis of a single sample^30^.

Our study also has several limitations. First, due to the use of aggregated genome-wide data, whether the effect of handgrip on coronary artery disease varies by sex or age cannot be examined, although truly causal effect is expected to be consistent. Previous cohort studies of grip strength and cardiovascular mortality showed mixed results on effect modification by sex, with one suggesting that grip strength was more predictive of mortality in men^31^, others found that the association was consistent in men and wome^3, 32^. Such discrepancies suggest that the association could be due to residual confounding. Second, while no obvious pleiotropy was reported for the 2 genetic variants used, the possibility of residual pleiotropy cannot be fully ruled out. Third, canalization may also influence the results. However, as canalization reflects compensatory mechanisms, it tends to bias the gene-exposure association towards the null^5^. The use of multiple genetic variants as instrumental variables may, to some extent, compensate this influence. Fourth, while the two selected SNPs were not in linkage disequilibrium with each other, it still possible that they are in linkage disequilibrium with SNPs that influence unknown risk factors for coronary artery disease. Fifth, some cohorts in the CHARGE consortium are also included in the CARDIoGRAMplusC4D consortium. Therefore the possibility of sample overlapping cannot be fully ruled out. This may have introduced bias in the results; however, given the sample size employed, this effect would likely be small since the CHARGE comprised <5% of the overall CARDIoGRAMplusC4D consortium^33^. Finally, given the small number of genetic variants employed in this study, further Mendelian randomization analyses using more SNPs identified from updated GWAS are warranted to replicate the current study.

In conclusion, this study provides evidence supporting a causal role for higher grip strength in lowering coronary artery disease risk. The findings offer a further rationale for physical activity or resistance training to maintain muscular strength in older age.
